# Supplementary material for: The loci of environmental adaptation in a model eukaryote
Source: Nat Commun. 2024 Jul 6;15:5672. doi: 10.1038/s41467-024-50002-y (PMC11227561; doi:10.1038/s41467-024-50002-y)
Supplement: Supplementary file 1 — Supplementary information [file 41467_2024_50002_MOESM1_ESM.pdf]

Supplementary information of "The loci of environmental adaptation in a model eukaryote" by P. Chen and J. Zhang

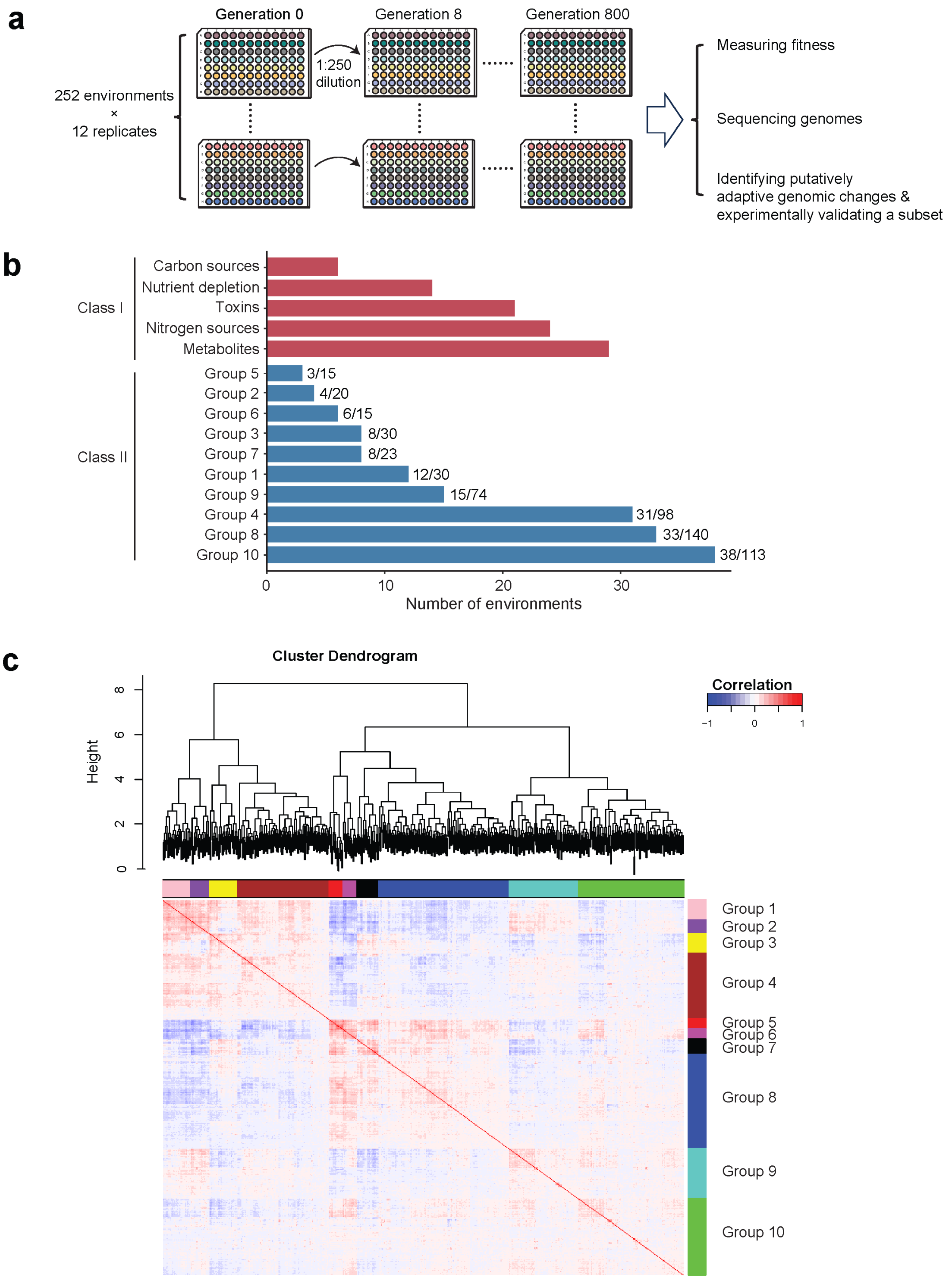

Supplementary Fig. 1. Study design. a, Schematics of yeast experimental evolution followed by downstream experiments and analyses. **b**, The environments used in experimental evolution. Class I includes 94 environments varying in carbon sources, nitrogen sources, nutrient depletion, toxins, and

metabolites. Class II includes 158 environments with druglike small molecules, separated into 10 groups. The numbers on the right side of a bar show the ratio of the number of environments used to the number available in the group. **c**, Ten groups of environments in Class II. We identified 558 commercially available chemicals and clustered them into 10 groups based on the similarities measured by the correlations in the growth rates of ~5,900 yeast gene deletion strains in the media containing these chemicals. In the heat map, each column/row represents one chemical. The vertical and horizontal color bars next to the heat map show the 10 groups of chemicals identified from the dendrogram when a cutoff of height = 4 is applied. See Methods for details. Source data are provided as a Source Data file.

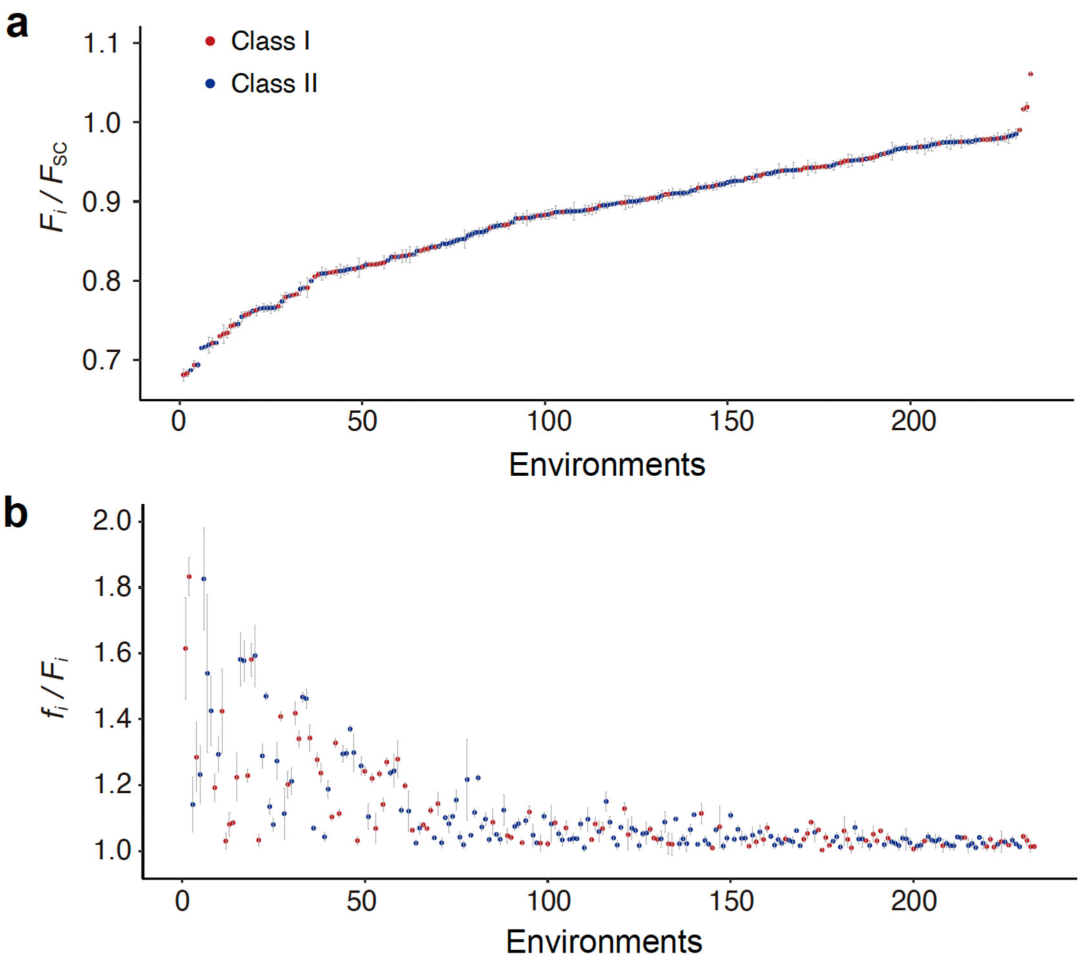

Supplementary Fig. 2. Fitness changes in experimental evolution. Same as Fig. 1ab, except that the two classes of environments in Supplementary Fig. 1b are marked with different colors. The extent of adaptation (f_i/F_i) is not significantly different between the two classes of environments (P = 0.47, two-tailed Wilcoxon rank-sum test). Source data are provided as a Source Data file.

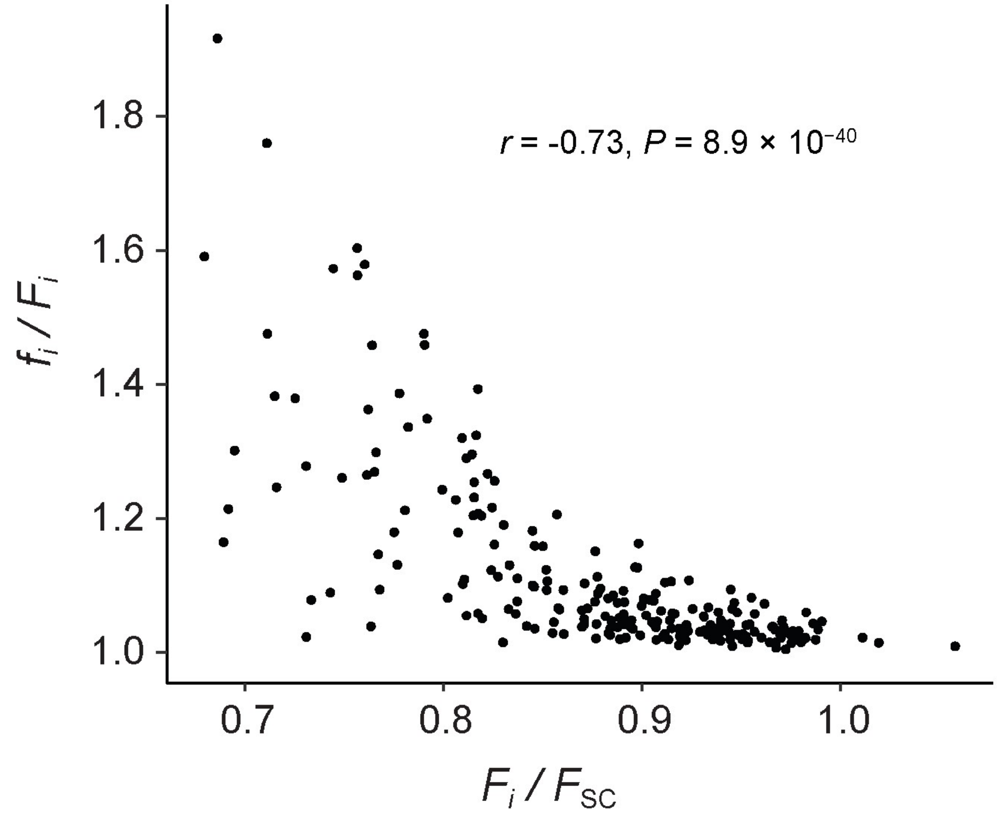

Supplementary Fig. 3. Negative correlation between the relative progenitor fitness in an environment (F_i/F_{SC}) and the extent of adaptation (f_i/F_i) in the environment. Same as Fig. 1c, except that F_i/F_{SC} is computed using the first two measurements of F_i while f_i/F_i is computed using the third measurement of F_i to avoid the non-independence between the values of the two axes in the figure. Source data are provided as a Source Data file.

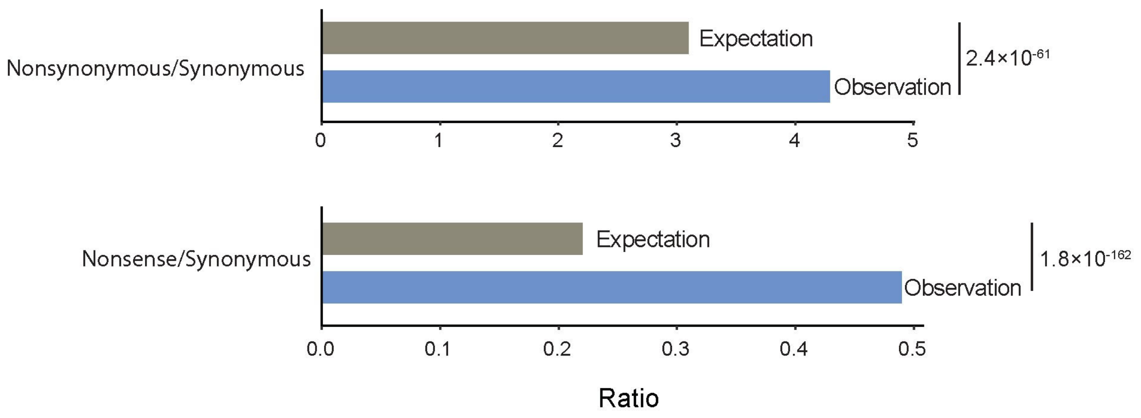

Supplementary Fig. 4. Expected and observed relative numbers of various types of coding substitutions. Same as Fig. 2b, except that the neutral expectation is based on the mutation spectrum in a rich medium rather than that estimated from the merged mutation accumulation data from seven different environments. Source data are provided as a Source Data file.

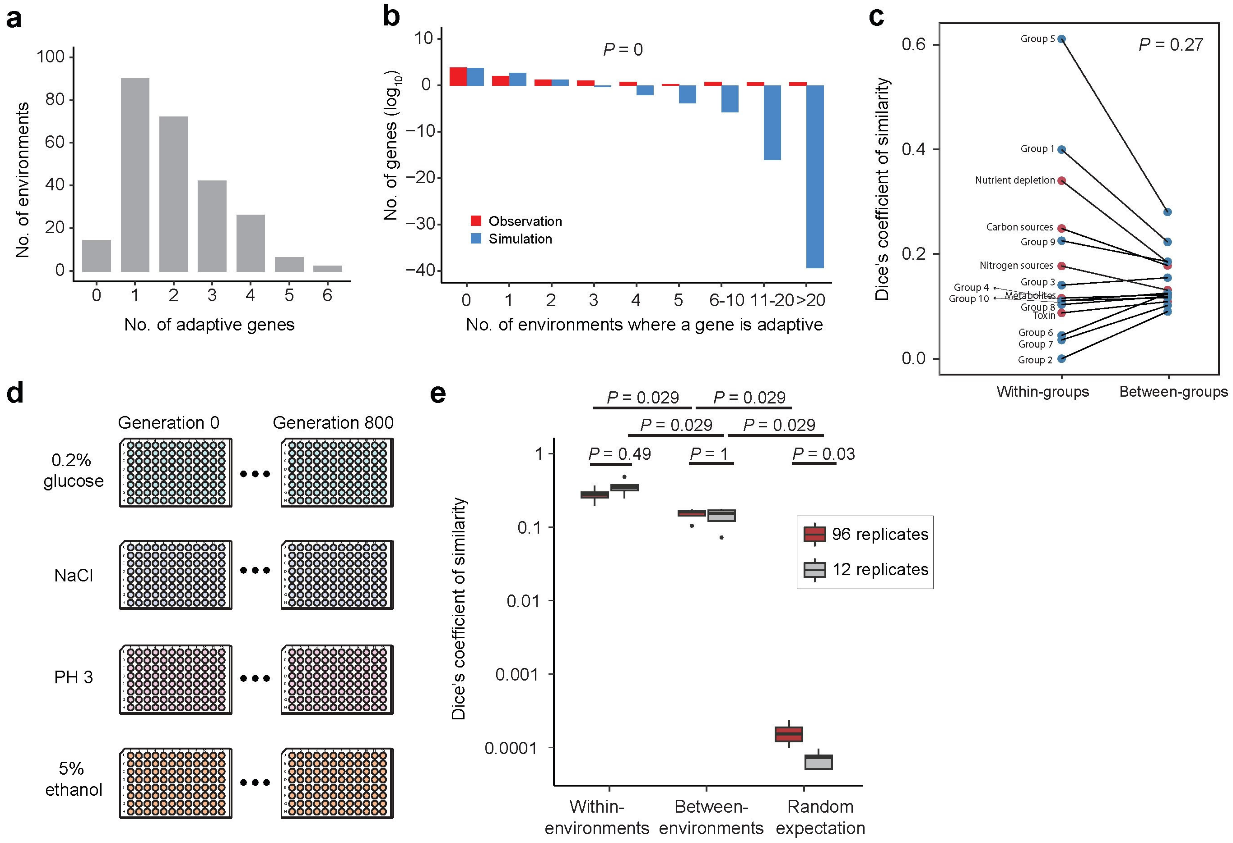

Supplementary Fig. 5. Statistics of putatively adaptive genes. a, Frequency distribution of the number of putatively adaptive genes per environment. **b**, Observed frequency distribution (red bars) of the number of environments where a gene is putatively adaptive, compared with a simulated Poisson distribution with the same mean (blue bars). *P*-value is from a one-tailed chi-squared test comparing the variance of the observed distribution with the corresponding Poisson variance. **c**, No significant difference in the mean Dice's coefficient of similarity in putatively adaptive genes between different environments in a group and those between groups. Red dots represent the five groups in Class I and blue dots represent the 10 groups in Class II in Supplementary Fig. 1b. In the within-groups column, a dot shows the mean Dice's coefficient between all pairs of environments within the focal group. In the between-groups column, a dot shows the mean Dice's coefficient between all environments in the focal group and all environments in other groups. *P*-value is from a two-tailed paired *t*-test for the 15 pairs of dots. **d**, Schematics of additional experimental evolution of 84 replicates in each of four environments. **e**, Comparison of Dice's coefficients from 96 and 12 replicates per environment for the four environments in **d**. Source data are provided as a Source Data file.

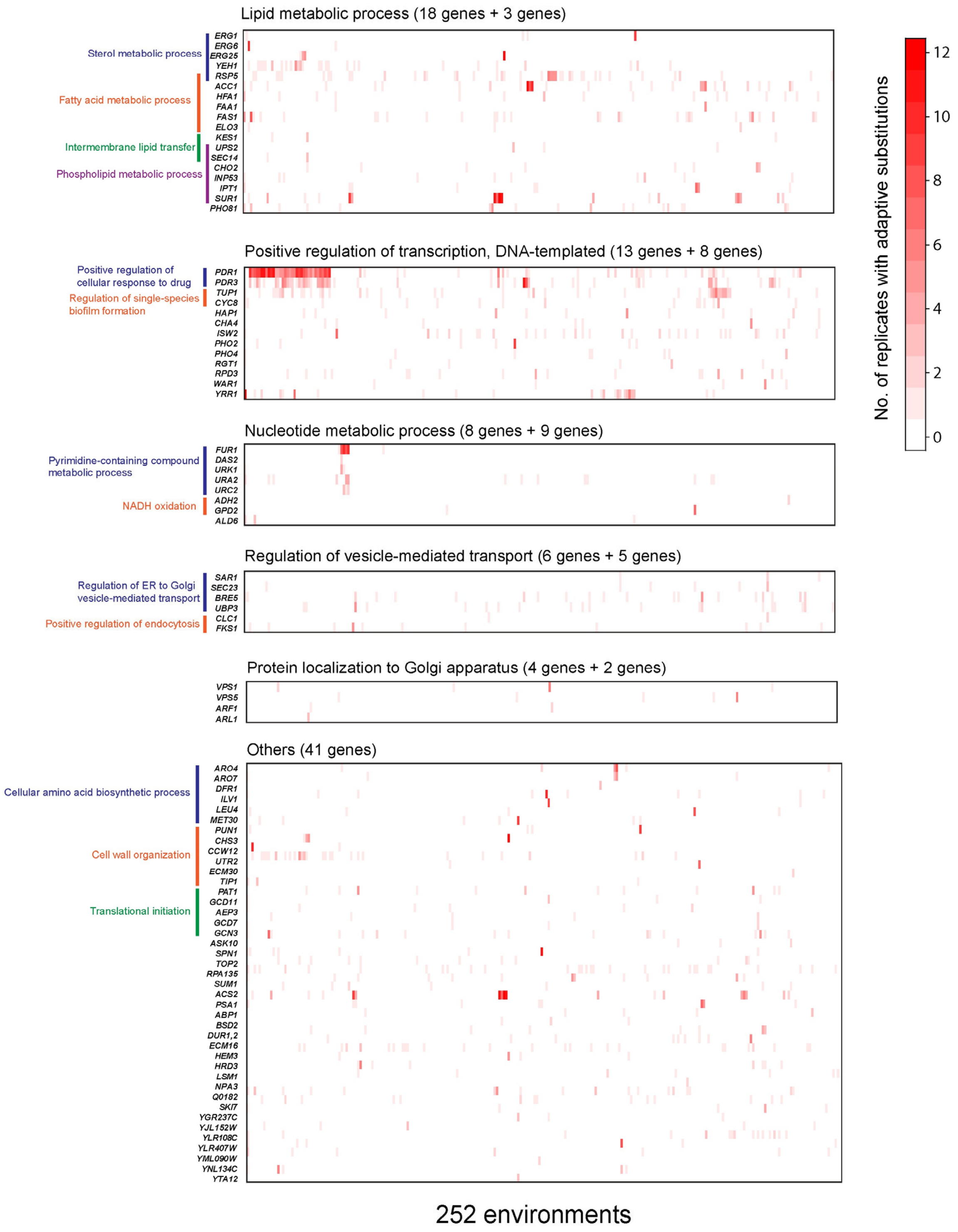

Supplementary Fig. 6. Additional enriched biological processes of the 149 putatively adaptive genes. Same as Fig. 5b, except that additional enriched biological processes are presented. Adaptive genes not belonging to significantly enriched biological processes are also listed. Source data are provided as a Source Data file.

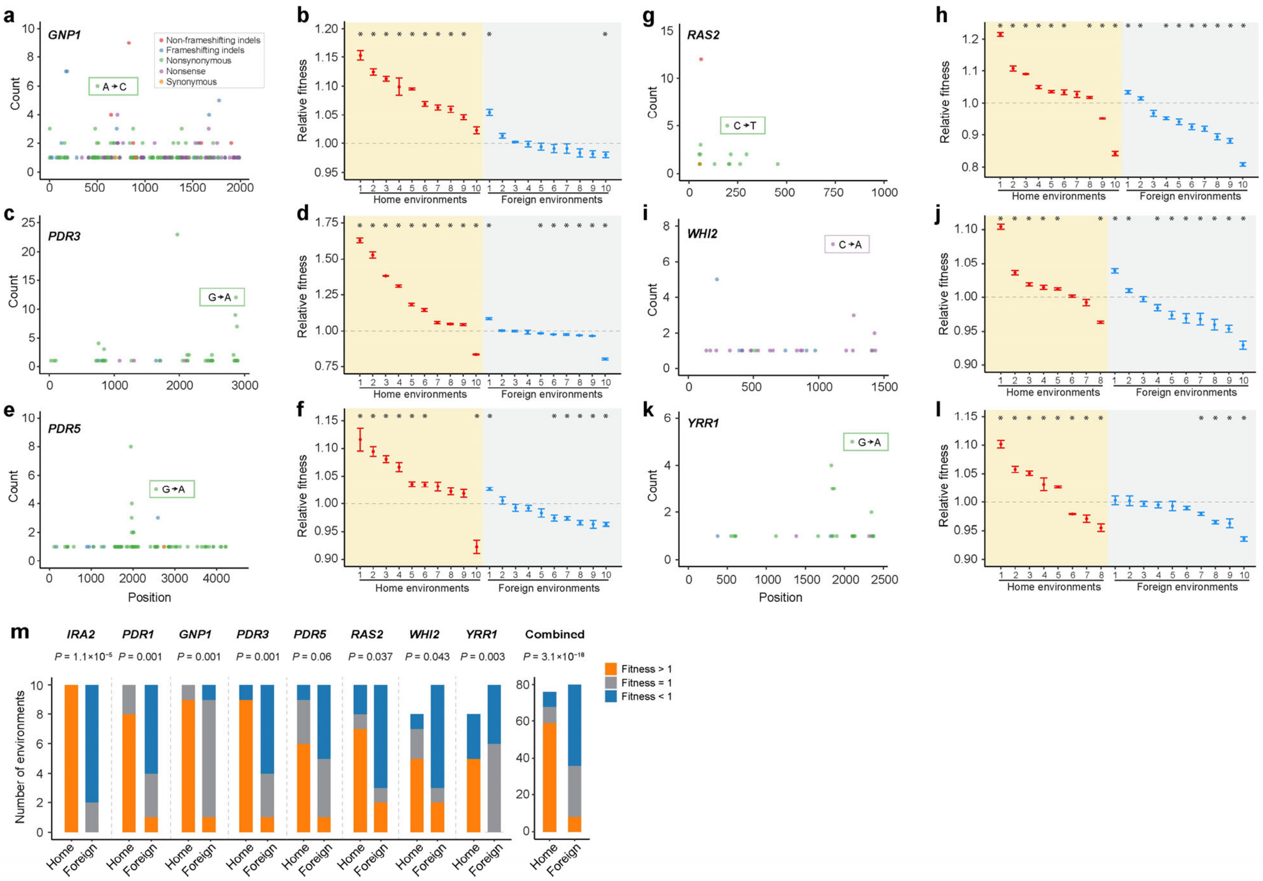

Supplementary Fig. 7. Fitness effects of six additional substitutions. a-l, Same as Fig. 6ab, except for six other substitutions. **m**, Numbers of home and foreign environments where the fitness of the mutated progenitor relative to the fitness of the progenitor is significantly higher than 1 (showing fitness >1 in the panel), not significantly different from 1 (fitness =1), and significant below 1 (fitness <1) for each mutant constructed. *P*-values are from one-tailed Fisher's exact tests (cases with relative fitness not significantly different from 0 are omitted). Source data are provided as a Source Data file.

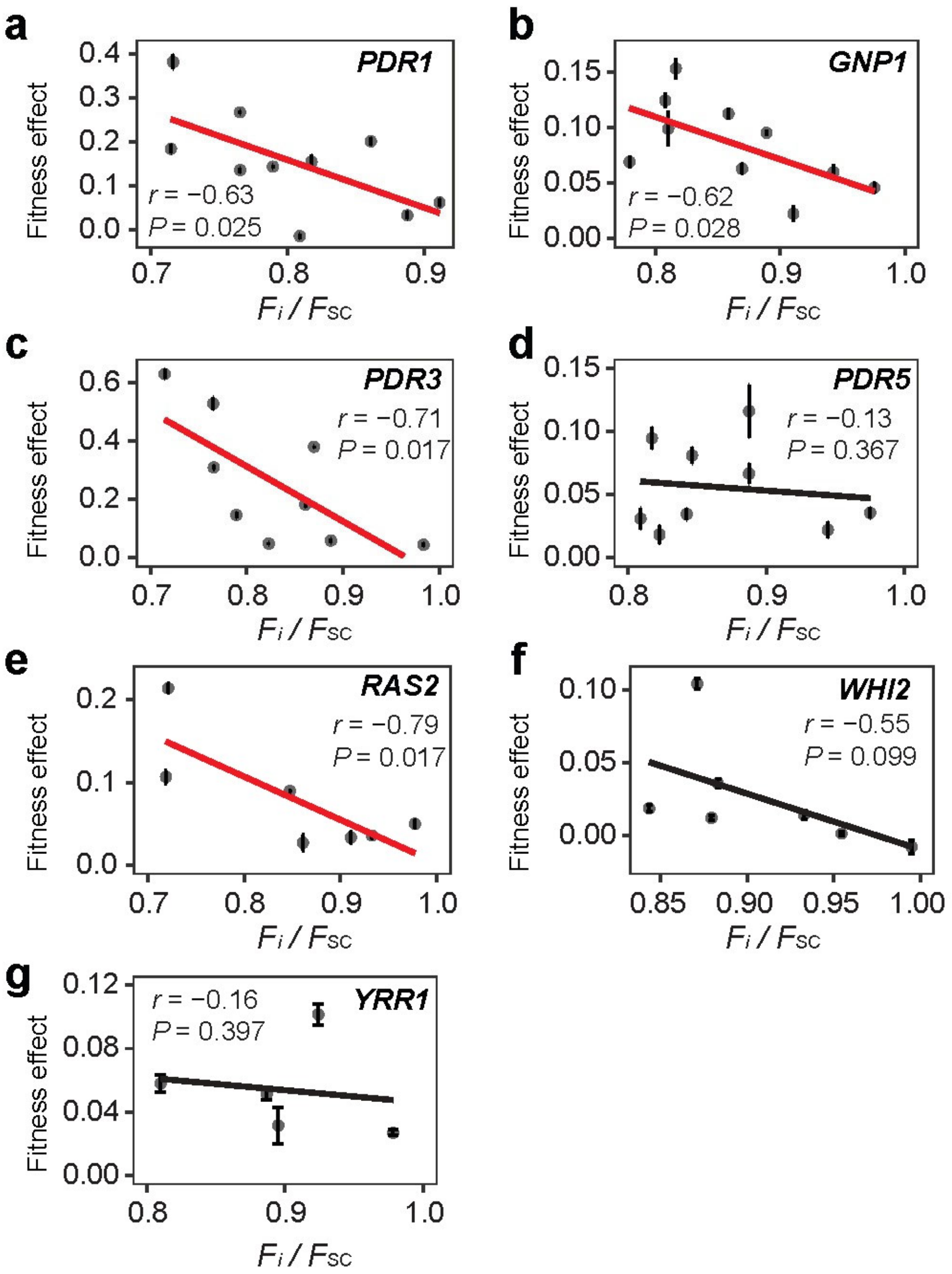

Supplementary Fig. 8. Larger effects of the same beneficial substitution in more stressful environments. Same as Fig. 6f except for seven other substitutions. Red and black lines respectively show significant and non-significant linear regressions. Source data are provided as a Source Data file.
