# Supplementary material for: The loci of environmental adaptation in a model eukaryote
Source: Nat Commun. 2024 Jul 6;15:5672. doi: 10.1038/s41467-024-50002-y (PMC11227561; doi:10.1038/s41467-024-50002-y)
Supplement: Supplementary file 3 — Description of Additional Supplementary Files [file 41467_2024_50002_MOESM3_ESM.pdf]

Description of Additional Supplementary Files

File Name: Supplementary Data 1

Description: The 252 media used in yeast experimental evolution.

File Name: Supplementary Data 2

Description: Seven environments with very high numbers of substitutions that are likely due to elevated mutagenesis rather than elevated selection.

File Name: Supplementary Data 3

Description: All observed single nucleotide substitutions and indels in yeast experimental evolution.

File Name: Supplementary Data 4

Description: Annotations of noncoding regions analyzed in this study.

File Name: Supplementary Data 5

Description: Putatively adaptive substitutions tested using genome editing.

File Name: Supplementary Data 6

Description: Primers for strain construction (gRNA target sequences bolded).
